# Toxicity and modulation of silver nanoparticles synthesized using abalone viscera hydrolysates on bacterial community in aquatic environment

**DOI:** 10.3389/fmicb.2022.968650

**Published:** 2022-08-30

**Authors:** Yue Zhang, Zhuan Yang, Jing Ni, Ying Ma, Hejian Xiong, Wenjie Jian

**Affiliations:** ^1^Engineering Research Center of the Modern Technology for Eel Industry, Ministry of Education, Fisheries College of Jimei University, Xiamen, China; ^2^College of Ocean Food and Biological Engineering, Jimei University, Xiamen, China; ^3^Institute of Respiratory Diseases, Xiamen Medical College, Xiamen, China

**Keywords:** silver nanoparticles, abalone viscera, zebrafish, bacterial community, aquaculture water

## Abstract

Polysaccharide decorated silver nanoparticles (AgNPs) are a new type of antibacterial agent in aquaculture, but their effects on the bacterial community structure in aquaculture water are still unknown. In this study, the primary hydrolysate from abalone (*Haliotis discus hannai*) viscera (AVH) was used to biosynthesize AVH-AgNPs by *in situ* reduction, and the crystallinity nature, size, morphology, and chemical composition were analyzed by high-resolution characterization techniques such as Ultraviolet–visible spectroscopy (UV–vis), X-rays diffraction (XRD), Transmission Electron Microscope (TEM), Dynamic light scattering (DLS), Zeta potential, inductively coupled plasma-optical emission spectrometry (ICP-OES) and Turbiscan stability index (TSI) values. Furthermore, the acute toxicity of AVH-AgNPs to zebrafish (*Danio rerio*) and their effects on bacterial community structure in fish culture water at low concentrations were studied. The results showed that the spherical AVH-AgNPs with an average diameter of 54.57 ± 12.96 nm had good stability, low toxicity, and good *in vitro* antibacterial activity. Within the experimental concentration range, all AVH-AgNPs treatments had decreased the bacterial diversity in zebrafish culture water to varying degrees. The bacteria with significantly decreased abundances were pathogenic or potential pathogenic, such as *Aeromonas veronii*, *Flavobacterium columnare*, and genera *Flectobacillus* and *Bosea*. The abundance of *Haliscomenobacter* sp. JS224, which might cause sludge swelling, also decreased significantly. On the other hand, the relative abundance of some bacterial taxa could remove xenobiotics (e.g., *Runella defluvii* and *Phenylobacterium*), control water eutrophication (*Sediminibacterium*), and reduce toxic algae proliferation (*Candidatus Intestinusbacter nucleariae* and *Candidatus Finniella*), increased significantly. Thus, the application of AVH-AgNPs in aquaculture water at low concentrations is relatively safe and has positive significance for improving the aquaculture environment. Also, AVH-AgNPs have good prospects in aquaculture.

## Introduction

Bacterial diseases are the most common diseases in aquaculture. At present, chemicals (e.g., antibiotics) are mainly used to control bacterial diseases. However, long-term exposure to antibiotics leads to bacterial resistance, environmental pollution, drug residues, and other public health hazards (Wang et al., 2020). Silver nanoparticles (AgNPs) are a new antibacterial product based on nanotechnology and have received increasing attention because of their high efficiency and broad-spectrum antibacterial ability (Yaqoob et al., 2020). The antibacterial mechanism of AgNPs is complex, and bacteria can hardly develop resistance against AgNPs (Guo et al., 2020). AgNPs have the prospect to replace the antibacterial drugs used in aquaculture and reduce the risk of drug-resistant bacteria and drug residues (Majeed et al., 2022).

AgNPs are mainly used to control diseases or improve the immunity of aquatic animals by adding these to the feed. Biosynthetic AgNPs have good antibacterial activity against *Streptococcus pneumonia*, *Staphylococcus aureus*, *Bacillus subtilis,* and various pathogenic *Vibrio* spp. *in vitro* (Oves et al., 2019, 2022; Jian et al., 2020). An appropriate amount of AgNPs added to the fodder has a protective effect. Immunomodulatory activities on juvenile Indian prawn (*Fenneropenaeus indicus*) (Vaseeharan et al., 2010) and giant tiger prawn (*Penaeus monodon*) (Kandasamy et al., 2013) infected by *Vibrio* spp. and the survival rate of white-leg shrimp (*Litopenaeus vannamei*) infected by *Vibrio parahaemolyticus* were improved (Alvarez-Cirerol et al., 2019). Low concentrations of AgNPs injected through the back of white-leg shrimp activated the immune system and enhanced the survival of white-leg shrimp infected with the white spot syndrome virus (WSSV; Ochoa-Meza et al., 2019).

A few studies have reported the bioeffects of AgNPs supplemented aquatic water. Rainbow trout (*Oncorhynchus mykiss*) infected with *Aeromonas salmonicida* did not show any mortalities or clinical signs after being soaked in 100 μg/ml of AgNPs solution (Shaalan et al., 2018). The low concentration of AgNPs can significantly improve the growth performance and stimulate the antioxidant enzyme activity of Nile tilapia (*Oreochromis niloticus*). In contrast, the higher concentration of AgNPs (>10 μg/l) may disrupt the growth performance and immune and antioxidant status and cause histopathological changes in gills, skin, liver, and intestines in Nile tilapia. Furthermore, the total bacterial count in culture water decreased with an increased concentration of AgNPs (Mabrouk et al., 2021). AgNPs have broad-spectrum antibacterial properties. However, their overall effects on the microbial community structure in aquaculture water have not been reported previously. Microorganisms in the aquatic environment play an important role in water quality control, substance metabolism, ecosystem stability, and disease control, which is crucial for maintaining a healthy culture environment (Blancheton et al., 2013). Understanding the effect of AgNPs on microbial community structure in aquaculture water is the prerequisite for the application of AgNPs in aquaculture.

During the chemical preparation process of AgNPs, reducing agents and surface modifiers need to be added, and the chemical toxicity of these adjuvant agents leads to safety problems for AgNPs (Ni et al., 2016). Using naturally derived macromolecules from plants, animals, and fungal cells for AgNPs synthesis reduces the toxicity and improves the stability of nanoparticles (Zafar et al., 2016; Ottoni et al., 2020). Previously, we synthesized polysaccharide-protein complex-silver nanoparticles (PSP-AgNPs) with high dispersion stability and high biosafety using PSP prepared from abalone (*Haliotis discus hannai*) viscera (Jian et al., 2019). Considering the complexity of PSP preparation, abalone viscera hydrolysate (AVH) obtained by primary enzymatic hydrolysis was used in this study as a capping agent and a stabilizer, and the AVH-AgNPs were successively achieved by thermal reduction. The aim of this study was to assess the acute toxicity of AVH-AgNPs on zebrafish (Danio rerio), and to investigate the influence of AVH-AgNPs on bacterial community structure in zebrafish aquaculture water, which will lay a foundation for the application of AVH-AgNPs in aquaculture purposes.

## Materials and methods

### AVH-AgNPs preparation and characterization

Abalone viscera was purchased from Xiamen Dao Zhiyuan Biotechnology Co., Ltd., Xiamen, Fujian, China. AVH was prepared according to the method described by Wang et al. (2015). The contents of protein and polysaccharides in AVH were 36.37% ± 0.41% and 32.04% ± 0.11%, respectively. The contents of fat, ash, and water were 8.11% ± 0.25%, 10.54% ± 0.16%, and 8.81% ± 0.02%, respectively.

The preparation procedures of AVH-AgNPs were described in a previous study (Jian et al., 2019). AVH was used as a reductant and a stabilizer. The reaction conditions were modified as follows: The final concentrations of AgNO_3_ and AVH in 100 ml of aqueous mixtures were 1.3 mM and 0.7 g/l, respectively. The mixture was then adjusted to pH 7.0 and left to react for 6 h at 100°C.

At first, the absorbance of AgNPs was determined by Ultraviolet–visible spectroscopy (UV-8000, Metash Instruments, China) in the wavelength range of 300–800 nm. The crystal structure of AVH-AgNPs was determined by BrukerAXS D8 Advance X-rays diffraction (XRD) (BrukerAXS D8 Advance, Bruker AXS, Germany), and their shapes were observed through a transmission electron microscope (TEM) (FEI talosf200s, Carl Zeiss AG, Germany). The size of nanoparticles and their distribution were measured by Dynamic light scattering (DLS), and the zeta-potential (ζ) of AVH-AgNPs was measured by Malvern ZS90 (Malvern Instruments, Malvern, United Kingdom). AVH-AgNPs were digested with a microwave, and the content of Ag in AVH-AgNPs was determined with inductively coupled plasma-optical emission spectrometry (ICP-OES) (Agilent Technologies 720, Santa Clara, CA, United States). In addition, the dispersion of AVH-AgNPs was evaluated by the multiple light scattering analyses (MLS). The Turbiscan stability index (TSI) was used to describe the results.

### Antibacterial activity of AVH-AgNPs against pathogenic bacteria

The antibacterial assessment was performed using two kinds of conventional indicator bacteria species, Gram-negative bacteria *Escherichia coli* ATCC352187 and Gram-positive bacteria *Staphylococcus aureus* ATCC25923, purchased from the Institute of Microbiology, Chinese Academy of Sciences, and two kinds of aquaculture pathogenic bacteria, *Aeromonas hydrophila* B11 isolated from diseased *Anguilla japonica* (Qin et al., 2014), and *V. parahaemolyticus* NPL1004 isolated from diseased *Pseudosciaena crocea* (Richardson; Yan et al., 2001). Minimum inhibition concentrations (MICs) were measured with twofold broth microdilution method (Ramli et al., 2017). With the initial concentration of 17.40 μg/ml, a total of eight double dilutions (17.40, 8.70, 4.35, 2.18, 1.09, 0.54, 0.27, and 0.14 μg/ml) of AVH-AgNPs were prepared. Each dilution was added with the same amount of 0.5 ml bacterial solution (1*10^5^ colony-forming unit (CFU)/ml), and the mixtures were incubated at the optimum temperature (37°C for *E. coli* and *S. aureus* and 28°C for *A. hydrophila* and *V. parahaemolyticus* for 24 h). Negative control (without bacteria) and positive control (with bacteria but no AVH-AgNPs) were also incubated following the same protocol. All the treatments and controls were repeated three times.

After cultivation, both observations with naked eyes and the measurement of absorbance at optical density 600 (OD600) were used for MIC determination. The MICs were the lowest concentration of AVH-AgNPs that completely inhibited bacterial growth. The cultures (100 μl) from the treatments without bacteria growth were plated on the solid nutrient broth medium (Mueller Hinton Broth, Huankai Microbial, Guangdong, China) and incubated for 24 h. The minimum bactericidal concentration (MBC) of AVH-AgNPs was determined by the lowest concentration with no or less bacterial growth (<5 colonies).

### Acute toxicity of AVH-AgNPs to zebrafish

One thousand zebrafish were purchased from Yudu Aquarium in Xiamen, Fujian Province, China. During the cultivation period, the water temperature was kept at 25°C ± 2°C, and the pH was 7 ± 0.2. Dissolved oxygen (DO) in the water was 7.9 ± 0.1 mg/l, and the light/dark period was 12/12 h. The experiment began after two weeks of adaptive cultivation.

Median lethal concentration (LC50) was determined according to the Organization for Economic Cooperation and Development (OECD) chemical test guide (Jochen, 2013). Seven culture water treatments with different concentrations of AVH-AgNPs (0, 100, 150, 200, 250, 300, and 350 μg/l) were designed, and each treatment had five parallels with a total of 35 culture boxes. A total of 350 healthy zebrafish were randomly selected and distributed into the 35 culture boxes with 10 fish per box (5 l water) for 96 h. At 24, 48, 72, and 96 h, the poisoning symptoms and mortality of zebrafish in the culture boxes were observed, and the dead individuals were removed to avoid polluting the culture water. The LC50 values of 96 h were calculated by the Probit analysis (Yang et al., 2003). The safe concentration (SC) of AVH-AgNPs for zebrafish was calculated according to the following formula: SC = 1/10 * LC50 (Yang et al., 2003).

### Effects of AVH-AgNP on bacterial community structure in aquaculture water

#### Experimental design

Based on the results of the acute toxicity experiment, four culture water treatments with different concentrations of AVH-AgNPs: 0 (WC group), 6.23 (WL group), 9.35 (WM group), and 18.70 μg/l (WH group), which corresponded to 0, 1/10, 1/20, and 1/30 of 96 h-LC50 values, were designed. Each treatment was repeated three times. A total of 600 zebrafish acclimated for two weeks were randomly and evenly distributed into 12 fish tanks (80 l) with 50 fish per tank, corresponding to four treatments with triplicates. The fish were fed three times a day. One-third of the water was changed every day to clean up the feces, and AVH-AgNPs were added at the same time to maintain a constant concentration of AVH-AgNPs in the water.

#### Sample collection and DNA extraction

Water samples were collected on the 30th day of the experiment. Culture water (300 ml) was taken from each fish tank and microorganisms in the water were enriched by filtration with a 0.22 μm Millipore membrane (PALL, Ann Arbor, MI, United States). Total DNA was extracted from the filter membranes with the TruSeqTM DNA Sample Prep kit (QIAGEN, Hilden, Germany) according to the manufacturer’s instructions.

#### Illumina sequencing and data analysis

Polymerase chain reaction (PCR) amplification was performed on the extracted DNA using the primers, including 338F (5′-ACT CCT ACG GGA GGC AGC AG-3′) and 806R (5′-GGA CTA CHV GGG TWT CTA AT-3′) of V3–V4 region of bacterial 16S rRNA gene. PCR-qualified DNA samples were sealed and sent to Shanghai Majorbio Bio-pharm Technology Co., Ltd., Shanghai, China, for Illumina MiSeq sequencing.

After quality control, the obtained sequences were assigned to different operational taxonomical units (OTUs) using the RDP classifier method based on 97% sequence identity (Wang et al., 2007). A *t*-test was performed to verify the significant difference. Sequence classification and community composition of each sample were identified by comparing with the Silva database (Release132).^1^ The pattern reflecting the relationship among different samples was analyzed by Principal Component Analysis (PCA). Heatmaps were drawn by clustering according to the similarity of abundance among species or samples. All data were calculated by Statistical Package for the Social Sciences (SPSS) 18 (SPSS, Chicago, IL, United States), analyzed by one-way analysis of variance (ANOVA), and verified by the Duncan test. *p* < 0.05 was considered a significant difference.

## Results

### Synthesis and characterization of AVH-AgNPs

**Figure 1:**
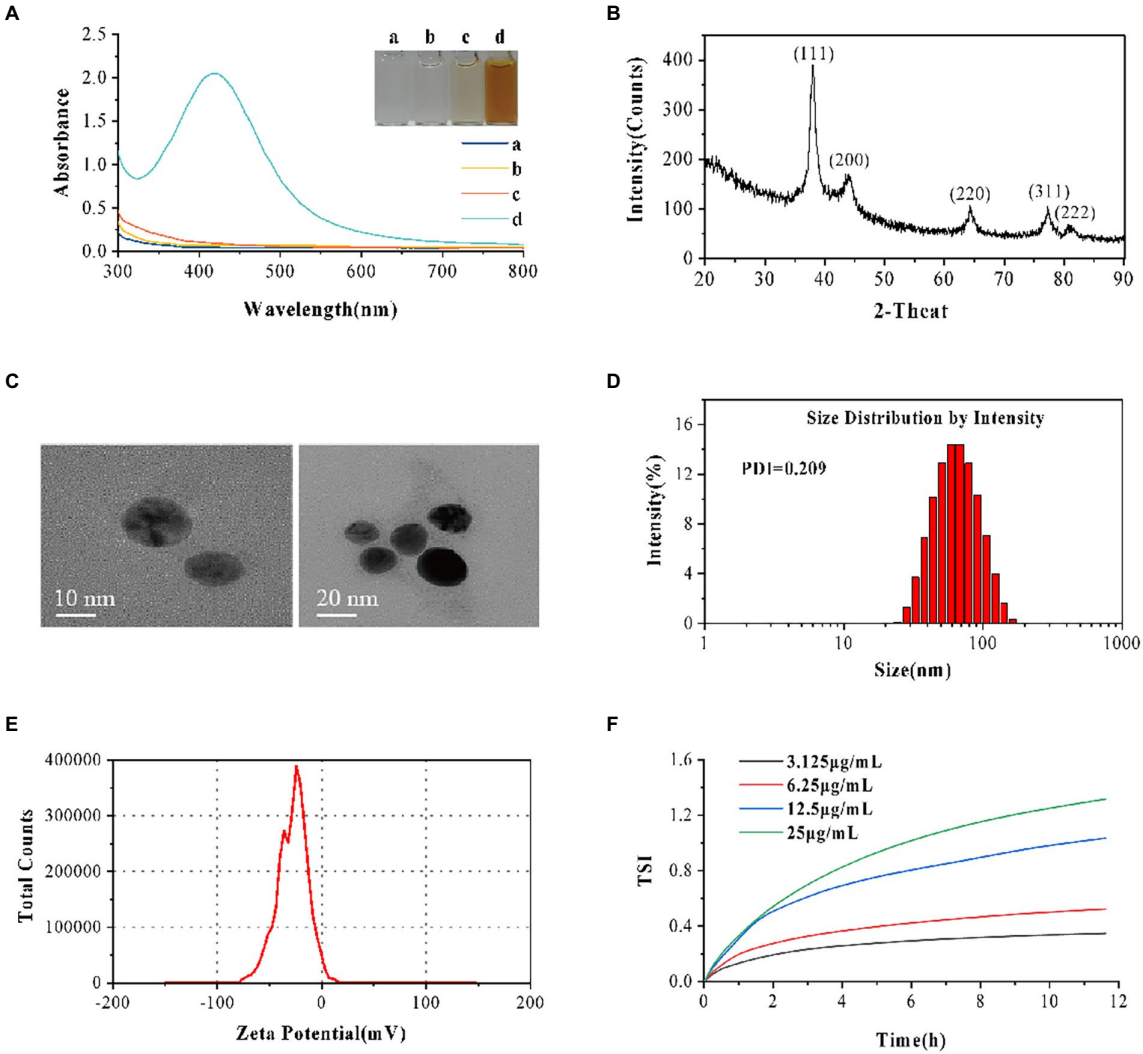
AVH-AgNPs characterization. **(A)** The Ultraviolet–visible spectroscopy (UV–vis spectra) demonstrated a strong absorption peak at 400–450 nm for AVH-AgNPs (a: AgNO_3_, b: H_2_O, c: AVH, d: AVH-AgNPs); **(B)** X-ray diffraction (XRD) analysis graph revealed the highest peak matching with the crystal planes of elemental silver; **(C)** The Transmission Electron Microscope (TEM) showed that AVH-AgNPs particles were spherical and well-dispersed; **(D)** Size distribution graph showed good dispersibility; **(E)** Zeta potential analysis observed −27.9 ± 4.3 mV with good stability and **(F)** Turbiscan stability index (TSI) adequately demonstrated the highly stable dispersion.

AVH-AgNPs characterizations are shown in Figure 1. The UV–vis demonstrated a strong absorption peak at 400–450 nm for AVH-AgNPs, without notice band for the water, AgNO_3,_ and AVH. Besides, the mixture of AVH-AgNPs was brown in color, while others were basically colorless and transparent, indicating that the AVH-AgNPs were successfully obtained (Figure 1A). From the XRD test, the prepared samples had obvious diffraction peaks at 2θ of 38.114, 44.298, 64.441, 77.395, and 81.538, respectively, which corresponded to the five different crystal planes of elemental silver (111), (200), (220), (311), and (222) respectively (Figure 1B). There were no other miscellaneous peaks in the XRD, indicating that the prepared AgNPs had high purity and good crystallization performance. The TEM of AVH-AgNPs (Figure 1C) showed that AVH-AgNPs particles were spherical and well-dispersed. The DLS diagram reflecting the size distribution of AVH-AgNPs is shown in Figure 1D. The size distribution was unimodal, with an average particle diameter of 54.57 ± 12.96 nm and a polymer dispersity index (PDI) value of 0.209, which indicated that the prepared AVH-AgNPs had good dispersibility without agglomeration. AVH-AgNPs colloid was stable, and its Zeta potential was −27.9 ± 4.3 mV (Figure 1E). ICP-OES analysis showed that the silver content in AVH-AgNPs was 9.00% ± 0.49%. Based on the MBC and MIC, we designed four concentrations of AVH-AgNPs at 3.125, 6.25, 12.5, and 25 μg/ml to detect the TSI over 12 h. The results showed that the TSI values of the four concentrations were 0.35, 0.52, 1.03, and 1.32, respectively (Figure 1F). The color of AVH-AgNPs did not change after 12 months of visual inspection at room temperature, indicating that AVH-AgNPs particles were well-dispersed in the aqueous solution with no obvious agglomeration.

### Antibacterial activities of AVH-AgNPs

The MICs and MBCs of the AVH-AgNPs against the four common pathogenic bacteria are shown in Table 1. AVH-AgNPs showed the strongest bacteriostatic and bactericidal activities against *E. coli*, with the MIC and MBC of 2.18 and 4.35 μg/ml, respectively, and the weakest antibacterial activities against *S. aureus*, with the MIC and MBC of 8.70 and 17.40 μg/ml, respectively. The antibacterial activities of AVH-AgNPs against the other two bacteria (*V. parahaemolyticus* and *A. hydrophila*) were similar, and the MIC and MBC of both strains were 4.35 and 8.70 μg/ml, respectively.

**Table 1:** *In vitro* antibacterial activity of AVH-AgNPs.

Concentration of AVH-AgNPs (μg/ml)	Bacterial strains			
	*Staphylococcus aureus*	*Escherichia coli*	*Vibrio parahaemolyticus*	*Aeromonas hydrophila*
MIC	8.70	2.18	4.35	4.35
MBC	17.40	4.35	8.70	8.70

### Acute toxicity of AVH-AgNPs to zebrafish

**Figure 2:**
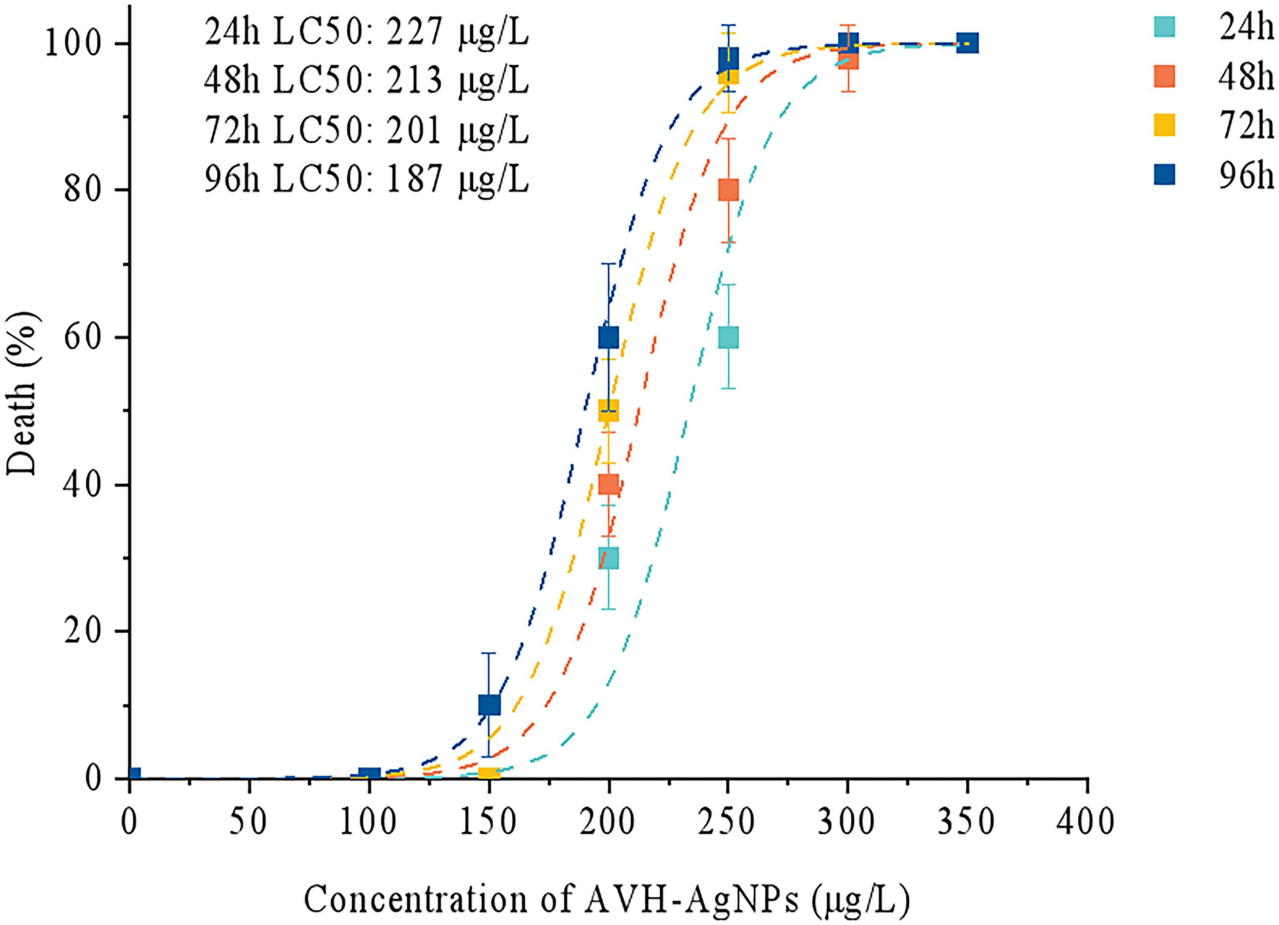
Median lethal concentration (LC50) of AVH-AgNPs on zebrafish, the LC50 values of AVH-AgNPs against zebrafish at 24, 48, 72, and 96 h were 227, 213, 201, and 187 μg/l, respectively.

Based on Probit analysis, the LC50 values of AVH-AgNPs against zebrafish at 24, 48, 72, and 96 h were calculated as follows: y = −31.901 + 13.538x, y = −32.808 + 14.082x, y = −45.904 + 19.924x, and y = −32.628 + 14.349x (where x is converted by logarithm with base 10.000), respectively, and the calculated LC50 values were 227, 213, 201, and 187 μg/l, respectively (Figure 2). Thus, the SC of AVH-AgNPs for zebrafish (calculated by 10 % of LC50 at 96 h) was 18.70 μg/l. In the following zebrafish exposure experiment, the SC, 1/2 SC, and 1/3SC corresponding to 18.70, 9.35, and 6.23 μg/l of AVH-AgNPs, respectively, were used to assess the effects of AVH-AgNPs on the microbial community structure in zebrafish farming water.

### Effects of AVH-AgNPs on bacterial community structure in zebrafish culture water

#### Sequence quality and alpha diversity

A total of 554,781 valid sequences were obtained from 12 culture water samples, with an average of 46231.75 ± 8812.65 sequences per sample and an average length of 415.15 ± 3.07 base pairs. A total of 29,000 sequences randomly selected from each sample were used for subsequent analysis to avoid analysis errors due to uneven sequencing. The coverages of all samples were more than 0.99, indicating that the sequencing depths could reflect the microbial diversity in the samples.

**Figure 3:**
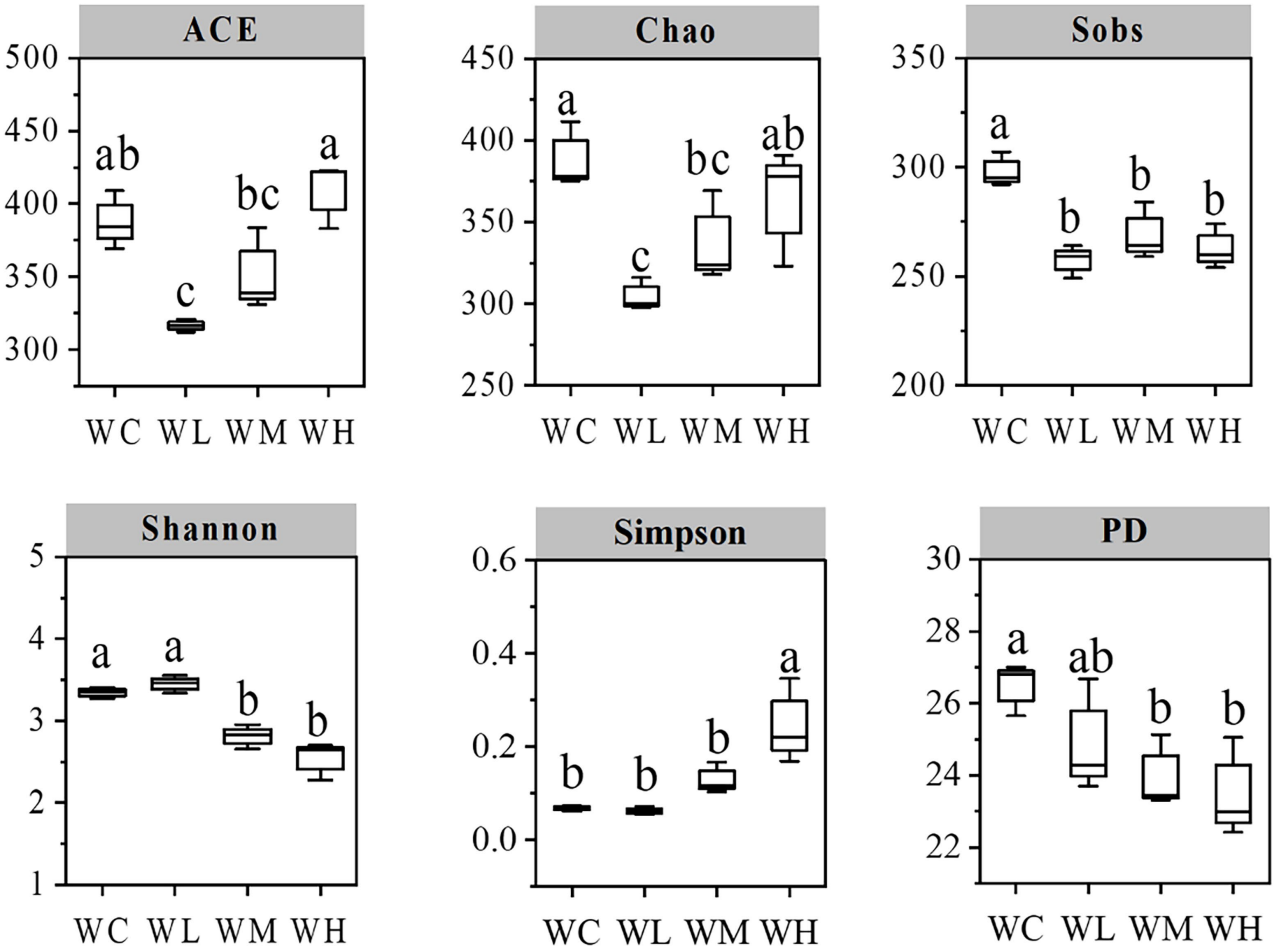
Index chart of microbial alpha diversity in aquaculture water. WC, WL, WM, and WH indicate four culture water treatments with different concentrations of AVH-AgNPs: 0, 6.23, 9.35, and 18.70 μg/l, respectively. Values with different superscripts within the same picture are significantly different (Duncan test, *p* < 0.05).

The diversity indexes of the samples are shown in Figure 3. Compared with the control (WC) group, the Sobs, ACE, and Chao indexes in the low concentration treatment group (WL group) decreased significantly (*p* < 0.05). Chao indexes of medium concentration treatment group (WM group), and Sobs, Shannon, and PD indexes of WM and high concentration treatment group (WH group) decreased significantly (*p* < 0.05). Furthermore, the Simpson index of the WH group increased significantly (*p* < 0.05). These results indicated that different concentrations of AVH-AgNPs treatment reduced the diversity of microorganisms in zebrafish culture water to different degrees, and the higher concentration had a more obvious effect.

#### PCA of microbial community

**Figure 4:**
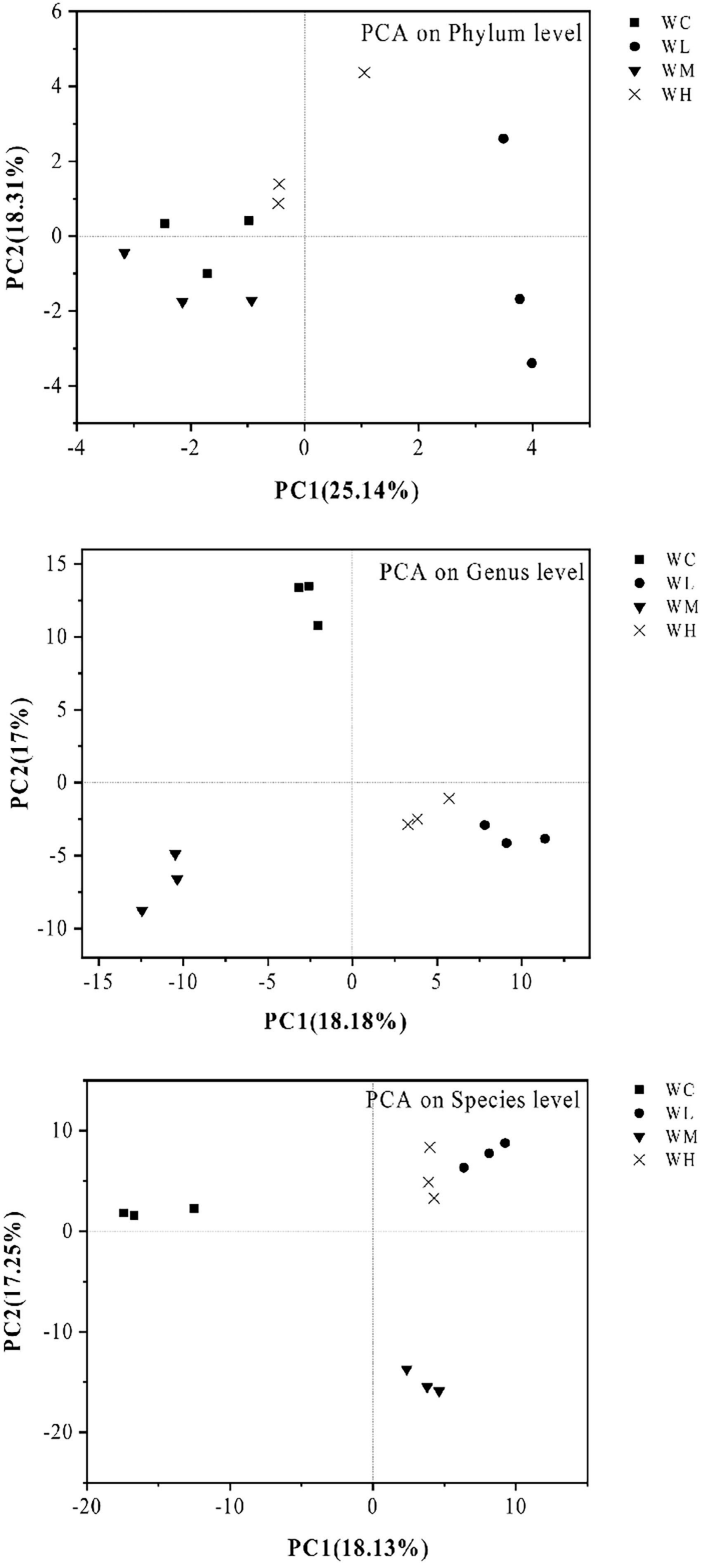
Principal component analysis (PCA) of microorganisms in aquaculture water of zebrafish. WC, WL, WM, and WH indicate four culture water treatments with different concentrations of AVH-AgNPs: 0, 6.23, 9.35, and 18.70 μg/l, respectively.

PCA results are shown in Figure 4. AVH-AgNPs treatment significantly affected the microbial community structure in zebrafish culture water. At the phylum level, the WL group was separated from the WC group obviously, while WM and WH groups were close to the WC group. At levels of genus and species, the samples from the same group clustered together, while the four groups (WC, WL, WM, and WH) were separated from each other. However, the distance between WL and WH groups was close, and the distance among other groups was further.

#### Effects of AVH-AgNPs on bacterial community structure in zebrafish culture water

A total of 25 phyla were detected in zebrafish culture waters, in which Proteobacteria, Actinobacteria, Bacteroidetes, and Acidobacteria were dominant and subdominant phyla, accounting for 26%, 12.88%, 12.88%, and 8.53%, respectively. The abundance of Chloroflexi, Chlamydiae, and Verrucomicrobia ranged from 1.03% to 1.78%. Eighteen phyla were also detected, including Fusobacteria, Armatimonadetes, Firmicutes, Spirochaetes, and Cloacimonetes, and they were all <1%.

At the genus level, a total of 359 genera were detected in zebrafish culture water, and 207 of these were classified into known genera. The dominant genera were *Rhodobacter* (13.35%) and *Flectobacillus* (5.98%), and the subdominant genera were *Flavobacterium*, *Polynucleobacter*, *hgcI_clade,* and *Legionella*, with an abundance ranged from 2% to 3.87%. Other genera with abundance greater than 1% were (in order of abundance) *Limnohabitans*, *Devosia*, *Phreatobacter*, *Aurantimicrobium*, and *Reyranella*. In addition, *Bosea*, *Aeromonas*, *Novosphingobium*, *Dinghuibacter*, *Methyloversatilis*, *Runella*, *Sediminibacterium*, etc., were detected, and their abundance ranged from 1% to 0.1%.

A total of 512 bacterial species were detected in zebrafish culture waters, of which 81 species were known. The known species with an abundance of more than 0.1% were *Polynucleobacter asymbioticus*, *Flavobacterium columnare*, *Aeromonas veronii*, *Haliscomenobacter* sp. JS224 and *Mycobacterium mucogenicum*. There are eight known species with abundance ranged from 0.1% to 0.01%, including *Edwardsiella ictaluri, Candidatus Kapabacteria* sp. 59–99*, Flavobacterium succinicans,* and *Candidatus Intestinusbacter nucleariae*.

**Figure 5:**
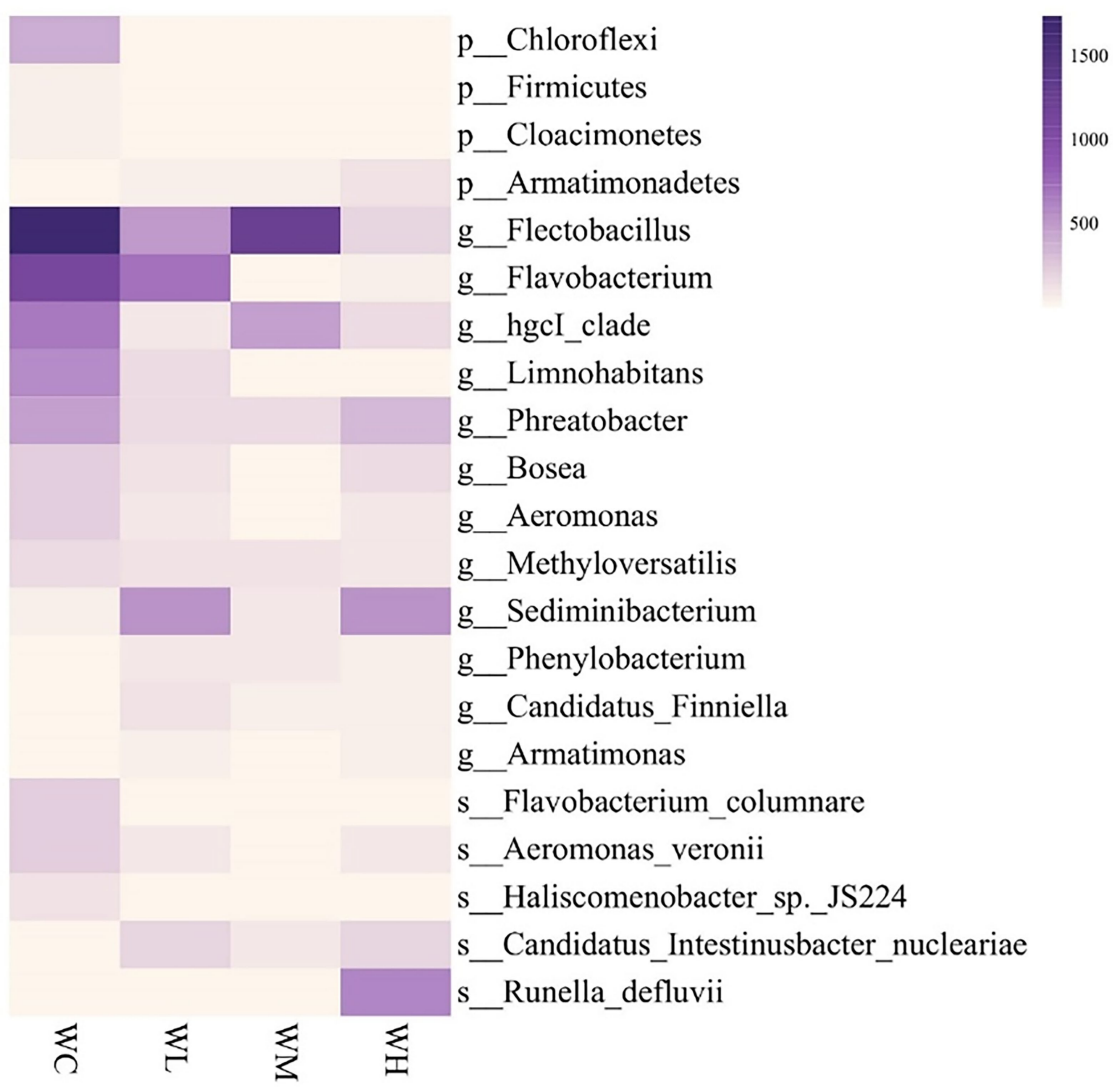
Heatmap analysis of top 50 identified bacterial taxa with significantly varied abundance in zebrafish culture water. WC, WL, WM, and WH indicate four culture water treatments with different concentrations of AVH-AgNPs: 0, 6.23, 9.35, and 18.70 μg/l, respectively. p-, g-, and s-represent classifications at phylum, genus, and species levels, respectively (Duncan test, *p* < 0.05).

The phyla, genera, and species that varied significantly in abundance among the top 50 bacterial taxa are shown in Figure 5. Compared with the control group, the abundance of Chloroflexi, Firmicutes, and Cloacimonetes decreased significantly, while the abundance of Armatimonadetes increased significantly in all treatment groups. At the genus level, after AVH-AgNPs treatment, *Flectobacillus*, *Flavobacterium*, *hgcI*_*clade*, *Limnohabitans*, *Phreatobacter*, *Bosea*, *Aeromonas,* and *Methyloversatilis* decreased significantly, while *Sediminibacterium*, *Phenylobacterium*, *Candidatus Finniella,* and *Armatimonas* increased significantly. At the species level, among the known species, *F. columnare*, *A. veronii*, and *Haliscomenobacter* sp. JS224 decreased significantly, while *Candidatus Intestinusbacter nucleariae* and *Runella defluvii* increased significantly in all treatments.

## Discussion

### Influence of enzymatic hydrolysis methods on physical characteristics of AVH-AgNPs

In this study, the AVH was used to prepare AVH-AgNPs. The average particle size of AVH-AgNPs was 54.57 ± 12.96 nm and was smaller than that of PSP-AgNPs (79.5 ± 10.4 nm) prepared by PSP from abalone viscera (Jian et al., 2019). Both AVH-AgNPs and PSP-AgNPs were spherical with Zeta potentials of −27.9 ± 4.3 mV and − 26.4 ± 2.7 mV, respectively, and could be stored for one year without precipitation. In addition, the TSI value was used to characterize the dispersion stability of AVH-AgNPs, a low TSI indicated a smaller particle size and higher stability (Agnese et al., 2018). In the present experiments, the TSI value of 25 μg/ml of AVH-AgNPs was 1.32, which is much smaller than the TSI values of the stable dispersions of iron or gold nanoparticles (up to 6) (Cupi et al., 2016; Qi et al., 2017). The TSI value adequately demonstrated the highly stable dispersion of AVH-AgNPs. These results indicated that spherical AgNPs with good dispersion could be prepared using AVH as a stabilizer and reducing agent, while the preparation protocol of AVH was simpler, and the cost was lower than those of PSP.

In previous studies, it took about 24–48 h to prepare AgNPs using biological materials to reduce silver ions (Ananthi et al., 2018; Kumar et al., 2018; Singh et al., 2018; Gitishree et al., 2019), while the spherical AgNPs could be synthesized within 6 h in this study, which shortened the preparation time of AgNPs.

### Antibacterial activity and acute toxicity of AVH-AgNPs

A previous study showed that AgNPs had bacteriostatic and bactericidal effects on Gram-positive *S. aureus* at a concentration of 0.625 mg/kg (Prashik et al., 2020). In the present study, the MIC and MBC of AVH-AgNPs against *S. aureus* were 8.70 and 17.40 μg/ml, respectively, which were lower than those of AgNPs synthesized by Prashik et al. (2020) and were also lower than those of PSP-AgNPs (Jian et al., 2019). AVH-AgNPs showed excellent antibacterial activity against Gram-negative bacteria. The MICs and MBCs against *E. coli*, *V. parahaemolyticus,* and *A. hydrophila* ranged from 2.18 to 4.35 μg/ml, and 4.35 to 8.70 μg/ml, respectively. All of these were lower than the MIC (6.25–12.5 μg/ml) and MBC (12.5 μg/ml) of the previously synthesized PSP-AgNPs (Jian et al., 2019, 2020), indicating that AVH-AgNPs had better antibacterial effects.

The 96 h-LC50 of AVH-AgNPs prepared in this study for adult zebrafish was 187 μg/l, which is higher than that of chemically synthesized AgNPs (0.0245 μg/ml; Rajan et al., 2018) and commercial AgNPs (0.028 μg/ml) (Masouleh et al., 2017). In addition, the LC50 value of AVH-AgNPs was higher than that of AgNPs synthesized with aqueous leaf extract of *Malva crispa* (0.1422 μg/ml) (Krishnaraj et al., 2016). These results indicated that the toxicity of AVH-AgNPs prepared in this study was lower. Zebrafish cultured in AVH-AgNPs (1/10 LC50 or lower) treated water for 30 d did not show any death and did not behave differently from the control zebrafish (without AVH-AgNPs treatment).

Previous studies showed that the antibacterial mechanism of AgNPs mainly included releasing silver ions, generating ROS, damaging cell structures, components, and important cell functions, etc. (Zhang et al., 2016; Yaqoob et al., 2020). The silver ion release from AgNPs in an aquatic environment is inevitable. In fact, it is generally accepted that silver ion release is the predominant antimicrobial mechanism of silver nanoparticles, which can be viewed from different perspectives (Zhang et al., 2016). Determining the certain concentrations of Ag^+^ release from AgNPs is complicated. Ag^+^ release can be affected by the particle size of AgNPs, the oxygen concentration in water, and other inorganic ions such as Cl^−^, etc. (Maryam et al., 2021). Zhang et al. (2016) proposed a two-stage model to predict silver ion release kinetics. However, more carefully-designed experimental work is necessary to determine the effects of size and other factors on nano silver dissolution/aggregation processes under varying aquatic environmental conditions. Further studies are needed to clarify the dissolution and Ag^+^ release of AVH-AgNPs in water environment to promote the application of nano-silver in aquaculture.

### Effects of AVH-AgNPs on bacterial community structure in zebrafish culture water

All the AVH-AgNPs treatments with different concentrations reduced the alpha diversity of bacteria in zebrafish culture water to different degrees, which was consistent with its antibacterial characteristics. Illumina sequencing analysis showed that the bacteria with significantly decreased abundance after AVH-AgNPs treatment were mainly associated with the disease. *A. veronii* is an emerging zoonotic and aquatic pathogen that threatens human health (Kang et al., 2018) and causes huge economic losses to aquaculture (Chao et al., 2021). *F. columnare*, widely distributed in the water environment, is a causative agent of columnaris disease and can infect a variety of commercial fish and cause high mortality. The gill necrosis and hemorrhagic disease caused by *F. columnare* infection are typical symptoms of fish columnaris disease (Maria et al., 2013). The abundance of both kinds of bacteria mentioned above accounted for 0.7% in the control group. In the AVH-AgNPs supplemented culture water, their abundance decreased significantly or below the detection limit in all treatments. *Flectobacillus* is a common genus in freshwater ecosystems and activated sludge. Its habitat distribution suggests the saprophytic lifestyle of this genus. *Flectobacillus roseus* within this genus is the causative agent of flectobacillosis (Adikesavalu et al., 2015). *Bosea* spp. are amoeba-resistant bacteria that colonize hospital water supplies and are the cause of acquired pneumonia in an intensive care unit (Khamis et al., 2003). In AVH-AgNPs treated zebrafish culture water, the abundance of these disease-related bacteria decreased significantly or had not been detected.

Compared with the control, the abundance of *Haliscomenobacter* sp. JS224, hgcl_clade, *Phreatobacter,* and *Methyloversatilis* also decreased significantly in AVH-AgNPs treatment groups. The biological function of *Haliscomenobacter* sp. JS224 is still unknown. The overproliferation of the genus *Haliscomenobacter* is an important cause of sludge swelling (Kowalska et al., 2016). Thus, the lower abundance of such bacteria had positive implications for maintaining a healthy farming environment. Previous studies showed that the abundance of *hgcl_clade* decreased with the increase of nutrient concentration (e.g., total nitrogen, TN; Liu et al., 2019), and *Phreatobacter* spp. were mainly isolated from ultra-pure water systems and preferred oligotrophic environments (Li et al., 2020). Also, *Methyloversatilis* spp. are versatile methyl users and can utilize a variety of nutrients and survive under oligotrophic conditions (Wu et al., 2021). The lower abundance of these bacteria in AVH-AgNPs treatments in this study might indicate a eutrophication trend in zebrafish culture water.

After adding AVH-AgNPs to zebrafish culture water, the relative abundance of some bacteria increased significantly, including *R. defluvii*, the genera *Phenylobacterium* and *Sediminibacterium*, and phylum Armatimonadetes. *R. defluvii* isolated from activated sludge can remove nutrients and organic matter from sewage (Lu et al., 2007). Genome analysis of a representative species of *Runella* showed that there were many genes associated with multi-drug efflux pumps (Keerthisinghe et al., 2019), which may be one of the reasons for its increased abundance after AgNPs treatment (Alex et al., 2012). *Phenylobacterium* prefers the phenyl in heterocyclic compounds as a carbon source and can degrade herbicide chloridazon (Eberspächer and Lingens, 2006). *Sediminibacterium* spp. were isolated from the sediments of the eutrophication reservoir (Pinto et al., 2018). Although the application value of *Sediminibacterium* is not known yet, it has great potential for environmental pollution control (Paes et al., 2016). The higher abundance of these bacteria in AVH-AgNPs treated aquaculture water has positive significance for controlling eutrophication, removing heteromorphic substances, and maintaining the health of the water.

After adding AVH-AgNPs, a few bacteria (abundance less than 0.1%), such as *Candidatus Intestinusbacter nucleariae* and *Candidatus Finniella*, also increased significantly. Both of them lived inside eukaryotic cells. *Candidatus Intestinusbacter nucleariae* was only detected in the cytoplasm of *Nuclearia delicatula* (algae that could prey on toxic cyanobacteria; Dirren and Posch, 2016). *Candidatus Finniella* is the novel endosymbiotic of viridiraptorid Amoeboflagellates (Cercozoa, Rhizaria; Hess et al., 2016). These endosymbiotic bacteria play important roles in host nutrition, defense, competition, and environmental adaptation (Liu et al., 2019). After the addition of AVH-AgNPs, the abundance of such bacteria in culture water increased, which may reduce the excessive proliferation of toxic cyanobacteria and maintain the health of the water environment.

After the addition of AVH-AgNPs to culture water, the abundance of many anaerobic bacteria, such as *hgcl_clade* (Ruprecht et al., 2021), Chloroflexi (Krzmarzick et al., 2012) and Cloacimonetes (Alalawy et al., 2021), decreased significantly, while the abundance of aerobic bacteria, such as Armatimonadota and *Armatimonas* (Liu et al., 2020) increased significantly, suggesting that the DO concentration in zebrafish culture water increased, which is also beneficial to the aquaculture system. In addition, the abundance of unknown species of Firmicutes and *Limnohabitans* decreased significantly after AVH-AgNPs treatment. The ecological significance of such changes in the abundance of these bacteria needs further study.

## Conclusion

The preparation method of AVH-AgNPs present in this study was simple, cheap, and time-saving. The spherical AVH-AgNPs showed good stability, low toxicity, and better *in vitro* antibacterial activity against all tested bacterial strains. The application of low concentrations of AVH-AgNPs in aquaculture water was relatively safe. All the AVH-AgNPs treatments decreased the bacterial diversity in zebrafish culture water to varying degrees. However, the decreased bacteria were mainly pathogenic or potentially pathogenic bacteria. Furthermore, the relative abundance of bacteria that could remove xenobiotics, control eutrophication, reduce the proliferation of toxic algae and maintain healthy farming environments increased significantly.

## Data availability statement

The raw data supporting the conclusions of this article will be made available by the authors, without undue reservation.

## Ethics statement

The animal study was reviewed and approved by the Animal Ethics Committee of Jimei University (Acceptance no. JMULAC201159).

## Author contributions

YZ and ZY performed the experiments and wrote the manuscript. JN organized the database and performed the statistical analysis. YM, HX, and WJ contributed to the conception and design of the study. All authors contributed to the article and approved the submitted version.

## Funding

This work was supported by the Natural Science Foundation of Fujian Province (grant no.: 2020 J01662), Fund of Engineering Research Center of the Modern Technology for Eel Industry, Ministry of Education (grant no.: RE202209), Xiamen Science and Technology Planning Project (grant no.: 2022CXY0307), the Program for Tackling Key Problems Jointly Funded by Fujian Provincial Health Commission and Education Department of Fujian Province (grant no.: 2019-WJ-40), and the program of Institute of Respiratory Diseases, Xiamen Medical College (grant no.: HXJB-14), China.

## Conflict of interest

The authors declare that the research was conducted in the absence of any commercial or financial relationships that could be construed as a potential conflict of interest.

## Publisher’s note

All claims expressed in this article are solely those of the authors and do not necessarily represent those of their affiliated organizations, or those of the publisher, the editors and the reviewers. Any product that may be evaluated in this article, or claim that may be made by its manufacturer, is not guaranteed or endorsed by the publisher.

## Footnotes

^1^ http://www.arb-silva.de
